# Preparando Pacientes e Otimizando Processos no Perioperatório das Cirurgias Cardíacas: Como Redesenhar os Fluxos de Assistência após a COVID-19

**DOI:** 10.36660/abc.20210484

**Published:** 2022-01-01

**Authors:** Omar Asdrúbal Vilca Mejia, Bruno Mahler Mioto, Gabrielle Barbosa Borgomoni, Juliana Mendanha Camilo, Danielle Misumi Watanabe, Sirlei Pereira Nunes, Vanessa Santos Sallai, Maraina Pegorini Libório de Lima, Jurema da Silva Herbas Palomo, Helenice Moreira da Costa, Elisandra Trevisan Arita, Maria Ignêz Zanetti Feltrim, Vera Coimbra, Roger Daglius Dias, Filomena Regina Barbosa Gomes Galas, José Otávio Costa Auler, Fabio Biscegli Jatene

**Affiliations:** 1 Universidade de Sao Paulo Faculdade de Medicina Hospital das Clinicas HCFMUSP Sao Paulo SP Brasil Instituto do Coracao (InCor), Hospital das Clinicas HCFMUSP, Faculdade de Medicina, Universidade de Sao Paulo, Sao Paulo, SP – Brasil; 2 Hospital Samaritano Paulista São Paulo SP Brasil Hospital Samaritano Paulista, São Paulo, SP – Brasil; 3 Harvard Medical School Boston Massachusetts EUA Harvard Medical School, Boston, Massachusetts – EUA

**Keywords:** Melhoria de Qualidade, Segurança do Paciente, Procedimentos Cirúrgicos Cardiovasculares, COVID-19, Recuperação Pós-Cirúrgica Melhorada

## Introdução

A falta de estratégias proativas de cuidados influenciam a fragmentação dos cuidados, desparametrização de processos e prolongamento de tempos hospitalares,^1^ enquanto evidências mostraram que protocolos multiprofissionais baseados em evidências executados por equipes sincronizadas, com padronização de processos, proatividade e cuidados centrados no paciente diminuem complicações, tempos e custos hospitalares.^2,3^ Neste contexto, cuidados baseados no conceito *Enhanced Recovery After Surgery* (ERAS) revolucionam os fluxos tradicionais.^4^ Diretrizes para cirurgia cardíaca foram publicadas recentemente,^5^ alcançando resultados encorajadores.^6,7^

Esta abordagem apontou eficiência e segurança na alta hospitalar em três dias após cirurgia cardíaca,^6,8^ algo promissor na era COVID-19, em que as filas cirúrgicas cresceram por adiamentos procedimentais, possibilitando maior número de atendimentos em menor tempo, redução do risco de contaminação e custos hospitalares.^9^

**Figura 1:**
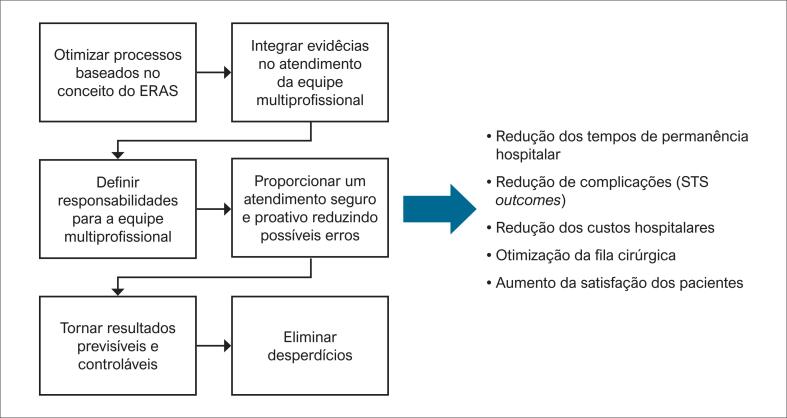
Objetivos da linha de cuidados Tempos Certos. ERAS: Enhanced Recovery After Surgery.

O Instituto do Coração, um dos maiores centros de cirurgia cardíaca,10 produziu um manual multiprofissional de cuidados baseado no conceito ERAS, otimizando processos através do preparo de pacientes para rápida recuperação após cirurgia cardíaca. A figura 1 apresenta os objetivos da implementação do fluxo Tempos Certos e seu possível impacto.

### Parecer

O manual Tempos Certos foi elaborado por representantes multiprofissionais do Instituto do Coração do Hospital das Clínicas da Faculdade de Medicina da Universidade de São Paulo, baseando-se em evidências no preparo de pacientes para um rápido retorno às atividades após cirurgia. O conceito é dinâmico, por isso o documento será revisado periodicamente.

### Critérios de Inclusão

Todo paciente programado para cirurgia cardíaca pode ser beneficiado pelos cuidados otimizados, quando coordenados por uma equipe multidisciplinar.

### Linha de Cuidados Tempos Certos

**Figura 2:**
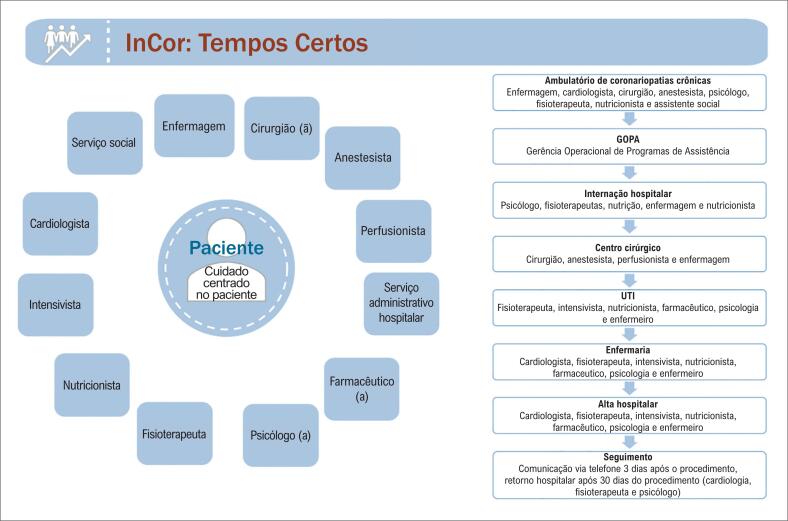
Equipes envolvidas no fluxo Tempos Certos.

A linha de cuidados transdisciplinar se inicia no ambulatório e é finalizada no seguimento após a alta hospitalar de cada paciente (Figuras 2 e 3).

**Figura 3:**
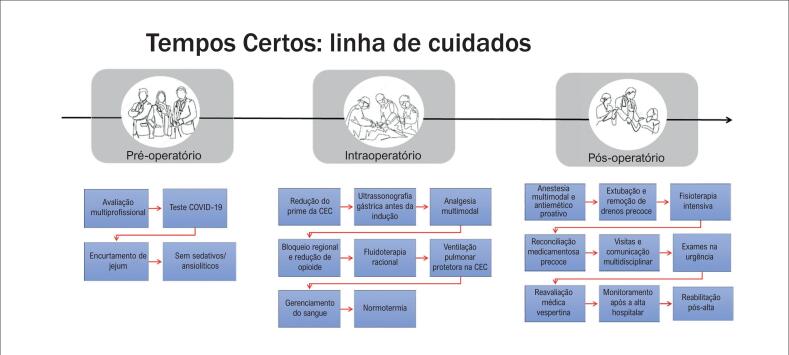
Mapa dos processos perioperatórios: Tempos Certos. CEC: circulação extracorpórea.

#### Ambulatório

1. Avaliação da adequação da indicação.
2. Avaliação odontológica, psicológica (questionário SF36 — qualidade de vida — e HADS — escala hospitalar de ansiedade e depressão), fisioterapêutica (medidas de repouso, teste de caminhada de seis minutos, ventilometria, *Short Physical Performance Battery* e reabilitação pré-operatória), nutricional (orientações sobre abreviação de jejum), anestésica, de enfermagem e serviço social.
3. Coleta de vigilância: COVID-19.

#### Pré-internação

1. Programar e confirmar a internação segundo orientações multiprofissionais.

#### Internação hospitalar

1. Pacientes eletivos: admissão 6 horas antes do procedimento.
2. Checagem da adesão ao protocolo no preparo prévio.
3. Refeição completa até 8 horas antes da indução anestésica.
4. Quebra do jejum 2 horas antes da cirurgia (líquido claro com maltodextrina, volume máximo 400 mL).
5. Não prescrever sedativos e/ou ansiolíticos.

#### Cirurgia

1. Identificação paciente Tempos Certos.
2. Redução do circuito da circulação extracorpórea (CEC) e da hemodiluição (perfusato <1000 mL).
3. Ultrassonografia gástrica pré-indução anestésica.
4. Analgesia multimodal (podendo ser usados: sulfato de magnésio, lidocaína, dextrocetamina e dexametasona antes da incisão, e dipirona ao final da cirurgia).
5. Sedação e bloqueio regional (eretor da espinha).
6. Redução do uso de opioides, podendo fazer uso de rocurônio/cisatracúrio, ketamina, dexdetomidina, propofol, isofluorano ou sevofluorane e antiemético.
7. Fluidoterapia guiada por metas (alvo balanço zero).
8. Índice bispectral e monitorização de bloqueio neuromuscular *train-of-four*.
9. Ventilação pulmonar 3-5 mL/Kg durante a CEC.
10. Drenagem torácica anterior.
11. Normotermia.
12. Glicemia <160 mg/dL.
13. Gerenciamento do sangue.
14. Transporte do paciente intubado para a unidade de terapia intensiva (UTI) sob efeito residual da anestesia, portando OXILOG e bomba de infusão com propofol ou precedex.

#### UTI

1. Identificação paciente Tempos Certos.
2. Anestesia multimodal (ketamina em PCA (*patient-controlled analgesia*), dipirona, dexametasona e tramal).
3. Antiemético preventivo.
4. Extubação em até 6 horas.
5. Pressão positiva contínua em vias aéreas (*continuous positive airway pressure* ou CPAP) por até 1 hora.
6. Reintrodução de ingestão oral (dieta líquida) quando houver lucidez (a partir de 2 horas após extubação).
7. Remoção dos drenos após redução da curva de sangramento (ultrassom confirma ausência de derrame).
8. Fisioterapia 6/6 horas: ausculta pulmonar e SpO2, estímulo à sedestação, exercícios respiratórios e motores, deambulação precoce, CPAP por 40 minutos.
9. Avaliação de enfermagem, incluindo avaliação *delirium* e dor: 1/1h até 12 horas de internação e 2/2 horas quando >12 horas.

#### Enfermaria

1. Identificação paciente Tempos Certos.
2. Reconciliação medicamentosa precoce.
3. Equipe multidisciplinar intensifica visitas e comunicação.
4. Fisioterapia 6/6 horas: ausculta pulmonar e SpO2, estímulo à sedestação, exercícios respiratórios e motores, deambulação precoce, CPAP por 40 minutos. Dia da alta: realizar medidas de repouso, teste da caminhada de seis minutos (meta >80%), ventilometria e *Short Physical Performance Battery* (*SPPB*).
5. Reavaliação médica no período da tarde (exames status urgência).
6. Reavaliação psicológica e reaplicação de questionários.
7. Aconselhamento educacional, nutricional e psicológico para alta hospitalar.

#### Seguimento (telessaúde ou presencial)

1. Monitoramento após 3 dias da alta hospitalar.
2. Reabilitação fisioterápica (sinais vitais, SPPB, teste de caminhada 6 minutos e capacidade vital pulmonar).
3. Reavaliação psicológica e aplicação questionários. Em caso de demanda emocional, reabilitação (psicoterapia breve focal).

### Possíveis contraindicações

Extubação precoce: sangramento importante, instabilidade hemodinâmica e respiratória e/ou falta de drive central respiratório.

Mobilização precoce: baixo débito cardíaco em uso de marcapasso epicárdico, instabilidade hemodinâmica (SVO2 <60, lactato alterado, noradrenalina 0,2 mcg/kg/min), delirium, sangramento >400 mL em 1 h >100 mL/h por 4 h seguidas, instabilidade respiratória – esforço respiratório.

### Considerações

Os sistemas de saúde têm avançando pouco frente a indústrias de alto desempenho. A chegada da COVID-19 impõe mudanças aceleradas para lidar com a nova realidade. A implementação dos conceitos de rápida recuperação, que já apresentavam resultados positivos na era pré-pandemia, inclusive em nosso cenário,^6^ mais do que nunca se tornaram necessárias para lidar com a demanda reprimida e ao mesmo tempo reduzir a exposição desnecessária do paciente ao ambiente hospitalar. O trabalho em equipe multidisciplinar, de forma sincronizada e harmônica, conseguiria adotar uma abordagem centrada no paciente, otimizando processos, melhorando a assistência e a segurança do paciente, assim como ampliando o acesso à saúde. Desta forma, conseguiríamos gerar valor no atendimento dos pacientes para a sustentabilidade dos programas de cirurgia cardíaca.
