# Supplementary material for: Preparando Pacientes e Otimizando Processos no Perioperatório das Cirurgias Cardíacas: Como Redesenhar os Fluxos de Assistência após a COVID-19
Source: Arq Bras Cardiol. 2022 Jan 1;118(1):110–4. [Article in Portuguese] doi: 10.36660/abc.20210484 (PMC8959051; doi:10.36660/abc.20210484)
Supplement: Translation

# Translation: Preparing Patients And Optimizing Processes In The Perioperative Period Of Cardiac Surgery: How To Redesign The Flow Of Care After COVID-19

**Authors:** Omar Asdrúbal Vilca Mejia, Bruno Mahler Mioto, Gabrielle Barbosa Borgomoni, Juliana Mendanha Camilo, Danielle Misumi Watanabe, Sirlei Pereira Nunes, Vanessa Santos Sallai, Maraina Pegorini Libório de Lima, Jurema da Silva Herbas Palomo, Helenice Moreira da Costa, Elisandra Trevisan Arita, Maria Ignêz Zanetti Feltrim, Vera Coimbra, Roger Daglius Dias, Filomena Regina Barbosa Gomes Galas, José Otávio Costa Auler, Fabio Biscegli Jatene

**Affiliations:** 1 São Paulo SP Brazil Instituto do Coração (InCor), Hospital das Clínicas HCFMUSP, Faculdade de Medicina, Universidade de São Paulo, São Paulo, SP – Brazil; 2 São Paulo SP Brazil Hospital Samaritano Paulista, São Paulo, SP – Brazil; 3 Boston Massachusetts USA Harvard Medical School, Boston, Massachusetts – USA

**Keywords:** Quality Improvement, Patient Safety, Cardiovascular Surgical Procedures, COVID-19, Enhanced Recovery After Surgery

## Introduction

The lack of proactive care strategies influences the fragmentation of care, deparametrization of processes and extension of hospital times,^1^ while evidence has shown that multidisciplinary protocols based on evidence executed by synchronized teams, with standardized processes, proactivity and patient-centered care decrease complications, hospital times and hospital costs.^2,3^ In this context, care based on the Enhanced Recovery After Surgery (ERAS) concept revolutionizes traditional flows.^4^ Guidelines for cardiac surgery were recently published,^5^ producing encouraging results.^6,7^

This approach showed efficiency and safety in hospital discharge within three days after cardiac surgery,^6,8^ which is promising in the COVID-19 era, in which surgical queues have grown due to procedural delays, enabling a greater number of visits in less time, reducing the risk of contamination and hospital costs.^9^

Instituto do Coração, a leading cardiac surgery center,^10^ produced a multiprofessional care guidebook based on the ERAS concept, optimizing processes by preparing patients for a quick recovery after cardiac surgery. Figure 1 presents the objectives of implementing the flow *Tempos Certos* and its possible impact.

**Figure 1:** *Objectives of the Tempos Certos care line. ERAS: Enhanced Recovery After Surgery*.

### Opinion

The *Tempos Certos* guidebook was created by multiprofessional representatives from Instituto do Coração do Hospital das Clínicas da Faculdade de Medicina da Universidade de São Paulo, based on evidence to prepare patients for a quick return to activities after surgery. The concept is dynamic, so the document will be revised periodically.

### Inclusion Criteria

Every patient scheduled for cardiac surgery can benefit from optimized care when coordinated by a multidisciplinary team.

### Tempos Certos Line of Care

The line of transdisciplinary care begins in the outpatient clinic and ends in the follow-up after each patient is discharged from the hospital (Figures 2 and 3).

**Figure 2:** *Teams involved in the Tempos Certos flow*.

**Figure 3:** Map of perioperative processes: Tempos Certos. CPB: cardiopulmonary bypass

#### Outpatient clinic

1. Assessment of appropriateness of the recommendation.
2. Dental and psychological assessment (SF36 questionnaire — quality of life — and HADS — hospital anxiety and depression scale), physiotherapy assessment (measures at rest, six-minute walk test, respiration, Short Physical Performance Battery and preoperative rehabilitation), nutritional assessment (guidance on fasting abbreviation), anesthetic and nursing evaluation, and social work.
3. Collection for surveillance purposes: COVID-19.

#### Pre-admission

1. Schedule and confirm admission according to multidisciplinary guidelines.

#### Hospital admission

1. Elective patients: admission 6 hours before the procedure.
2. Checking adherence to the preparation protocol.
3. Full meal up to 8 hours before anesthetic induction.
4. Fasting break 2 hours before surgery (clear liquid with maltodextrin, maximum volume 400 mL).
5. Do not prescribe sedatives and/or anxiolytics.

#### Surgery

1. Identification of *Tempos Certos* patient.
2. Reduced cardiopulmonary bypass circuit (CBC) and hemodilution (perfusate <1000 mL).
3. Anesthetic pre-induction gastric ultrasound.
4. Multimodal analgesia (the following can be used: magnesium sulfate, lidocaine, dextroketamine and dexamethasone before the incision, and dipyrone at the end of surgery).
5. Sedation and regional block (erector spinae).
6. Reduction of opioids. Rocuronium/cisatracurium, ketamine, dexdetomidine, propofol, isoflurane or sevoflurane and antiemetic can be used.
7. Goal-guided fluid therapy (zero balance target).
8. Bispectral index and monitoring train-of-four neuromuscular blockade.
9. Pulmonary ventilation 3–5 mL/kg during cardiopulmonary bypass (CPB).
10. Anterior thoracic drainage.
11. Normothermia.
12. Blood glucose <160 mg/dL.
13. Blood management.
14. Take the intubated patient to the intensive care unit (ICU) under residual effects of anesthesia, carrying OXILOG and infusion pump with propofol or precedex.

#### ICU

1. Identification of *Tempos Certos* patient.
2. Multimodal anesthesia (ketamine in PCA (patient-controlled analgesia), dipyrone, dexamethasone and tramal).
3. Preventive antiemetic.
4. Extubation within 6 hours.
5. Continuous positive airway pressure or CPAP for up to 1 hour.
6. Reintroduction of oral intake (liquid diet) when patient is conscious (from 2 hours after extubation).
7. Removal of drains after reduction of the bleeding curve (ultrasound confirms no effusion).
8. Physiotherapy 6/6 hours: pulmonary auscultation and SpO_2_, patient encouraged to sit, respiratory and motor exercises, early ambulation, 40-minute CPAP.
9. Nursing assessment, including delirium and pain assessment: 1/1 h up to 12 hours of hospitalization and 2/2 hours when >12 hours.

#### Ward

1. Identification of *Tempos Certos* patient.
2. Early drug reconciliation.
3. Multidisciplinary team intensifies visits and communication.
4. Physiotherapy 6/6 hours: pulmonary auscultation and SpO_2_, patient encouraged to sit, respiratory and motor exercises, early ambulation, 40-minute CPAP. Day of discharge: taking measurements at rest, six-minute walk test (goal >80%), respiration and Short Physical Performance Battery (SPPB).
5. Medical assessment in the afternoon (urgent tests).
6. Psychological reassessment and re-application of questionnaires.
7. Educational, nutritional and psychological counseling for hospital discharge.

#### Follow-up (by telephone or in person)

1. Monitoring 3 days after hospital discharge.
2. Physical therapy rehabilitation (vital signs, SPPB, 6-minute walk test and vital pulmonary capacity).
3. Psychological reassessment and application of questionnaires. In case of emotional demand, rehabilitation (brief focal psychotherapy).

### Potential contraindications

Early extubation: major bleeding, hemodynamic and respiratory instability and/or lack of central respiratory drive.

Early mobilization: low cardiac output using an epicardial pacemaker, hemodynamic instability (SVO_2_ <60, altered lactate, norepinephrine 0.2 mcg/kg/min), delirium, bleeding >400 mL in 1 h >100 mL/h for 4 h in a row, respiratory instability – respiratory effort.

### Comments

Health systems have made little progress compared to high-performance industries. The outbreak of COVID-19 requires fast changes to deal with the new reality. The implementation of rapid recovery concepts, which already had positive results in the pre-pandemic era, including in our scenario,^6^ became, more than ever, necessary to deal with the unmet demand while reducing the unnecessary exposure of patients to the hospital setting. Multidisciplinary teamwork conducted in a synchronized and harmonious way would be able to adopt a patient-centered approach, optimizing processes, improving patient care and safety, and expanding access to healthcare. This way, we would be able to generate value in patient care for the sustainability of cardiac surgery programs.
